# Protocol for spatial prediction of soil transmitted helminth prevalence in the Western Pacific region using a meta-analytical approach

**DOI:** 10.1186/s13643-024-02469-5

**Published:** 2024-02-06

**Authors:** Beth Gilmour, Kingley Wangdi, Angela Cadavid Restrepo, Tsheten Tsheten, Matthew Kelly, Archie Clements, Darren Gray, Colleen Lau, Fe Esperanza Espino, Chona Daga, Vanessa Mapalo, Susana Vaz Nery, Adam Bartlett, Eyob Alemayehu Gebreyohannes, Kefyalew Addis Alene

**Affiliations:** 1 School of Population Health, Faculty of Health Sciences, Curtin University, Kent St, Bentley WA, Western Australia 6102 Australia; 2 Geospatial and Tuberculosis Research Team, Telethon Kids Institute, West Perth, Western Australia Australia; 3 Australia National University, Canberra, Australia; 4 The University of Queensland, Brisbane, Australia; 5 Queen’s University, Belfast, Northern Ireland; 6 Research Institute for Tropical Medicine, Muntinlupa, Philippines; 7 The Kirby Institute, University of New South Wales, Kensington, Australia; 8 University of South Australia, Adelaide, Australia

**Keywords:** Soil transmitted helminth, STH, Western Pacific, Spatial prediction mapping

## Abstract

**Background:**

Soil transmitted helminth (STH) infections are estimated to impact 24% of the world’s population and are responsible for chronic and debilitating morbidity. Disadvantaged communities are among the worst affected and are further marginalized as infection prevalence fuels the poverty cycle. Ambitious targets have been set to eliminate STH infections, but accurate epidemiological data will be required to inform appropriate interventions. This paper details the protocol for an analysis that aims to produce spatial prediction mapping of STH prevalence in the Western Pacific Region (WPR).

**Methods:**

The protocol follows the Preferred Reporting Items for Systematic Review and Meta-Analysis Protocol (PRISMA-P) guidelines. The study design will combine the principles of systematic review, meta-analysis, and geospatial analysis. Systematic searches will be undertaken in PubMed, Scopus, ProQuest, Embase, and Web of Science for studies undertaken post 2000, to identify surveys that enable the prevalence of human STH infection within the WPR to be calculated. Covariate data for multivariable analysis will be obtained from publicly accessible sources. Survey data will be geolocated, and STH prevalence and covariates will be linked to produce a spatially referenced dataset for analysis. Bayesian model-based geostatistics will be used to generate spatially continuous estimates of STH prevalence mapped to a resolution of 1 km^2^. A separate geospatial model will be constructed for each STH species. Predictions of prevalence will be made for unsampled locations and maps will be overlaid for each STH species to obtain co-endemicity maps.

**Discussion:**

This protocol facilitates study replication and may be applied to other infectious diseases or alternate geographies. Results of the subsequent analysis will identify geographies with high STH prevalence’s and can be used to inform resource allocation in combating this neglected tropical disease.

**Trial registration:**

Open Science Framework: osf.io/qmxcj.

**Supplementary Information:**

The online version contains supplementary material available at 10.1186/s13643-024-02469-5.

## Background

Neglected tropical diseases (NTDs) are a disparate group of 20 diseases that have a devastating impact upon the lives of more than one billion people [1]. Soil transmitted helminth (STH) infections are the most widespread NTD and are estimated to impact 24% of the world’s population [2, 3].

Multiple species of STH, including *Ascaris lumbricoides* (roundworms), *Trichuris trichiura* (whipworms) and *Necator americanus*, *Ancylostoma duodenale* and zoonotic *Ancylostoma ceylanicum*, *Ancylostoma caninum*, and *Ancylostoma braziliense* (hookworms), are typically classified as a group due to diagnostic and treatment similarities [2]. This group of STHs prevail in the tropics and subtropics and have their greatest impact on disadvantaged communities where hygiene and sanitation are inadequate [2]. *Strongyloides stercoralis* and *Strongyloides fuelleborni* (threadworms) are another pathogenic STH of significance to human health, which are differentiated by an auto-infective capability within the lifecycle [4, 5].

STH infections result in chronic and debilitating morbidity, the extent of which is relative to the worm burden and influenced by host age and immunity [6]. Symptoms of STH infection include malnutrition, malaise, impaired physical and cognitive development, anemia, and intestinal obstruction [2, 7]. Symptoms of STH infection are often hard to identify due to the impact of poverty, malnutrition, and comorbidities which are common among those worst affected [8]. A key World Health Organization (WHO) STH control strategy is the administration of anthelmintic chemotherapy to at-risk population groups living in endemic areas [2]. At-risk populations include preschool children (PSAC), school age children (SAC), women of reproductive age, and personnel undertaking high-risk occupations [2]. The WHO recommends annual administration of anthelmintic chemotherapy to at risk populations where community infection prevalence exceeds 20% and bi-annual chemotherapy where prevalence > 50% [9].

“Ending the neglect to attain the Sustainable Development Goals; a road map for neglected tropical diseases 2021–2030” [10] is a World Health Organization (WHO) roadmap that aims to strengthen the response to eliminating NTDs [11]. Milestones within this roadmap include the elimination of STH infection as a public health problem in 96 countries by 2030 [10]. STH infection is defined as a public health problem, when the prevalence of moderate-to-heavy intensity infections > 2% in PSAC and SAC [12]. The roadmap approach is based on three pillars, including Pillar I, which seeks to accelerate programmatic actions through a thorough understanding of disease epidemiology [13].

This study aims to evaluate the prevalence of STH infections within the Western Pacific Region (WPR) by combining the principles of systematic review, meta-analysis, and geospatial analysis. This analytical approach has been shown to provide a cost-effective solution to determining STH distribution in sub-Saharan Africa [14], South America [15, 16], and South-East Asia [17]. but to our knowledge, this approach has not been applied to the WPR. Understanding infection prevalence is key to informing interventions, such as mass drug administration (MDA) which is the primary focus of the WHO STH control strategy [18].

This study aims to evaluate the prevalence of STH infections within the WPR and is designed to inform the prioritization of resources to address STH burden. The specific objectives of the study include the production of spatial prediction of STH prevalence and the evaluation of the spatial co-distribution and co-infection of ascariasis, trichuriasis, hookworms, and strongyloidiasis in the WPR. The environmental and climatic factors that influence the spatial distribution of STH infection within the WPR, will also be identified.

## Methods

### Data sources and search strategy

This protocol follows the Preferred Reporting Items for Systematic Review and Meta-Analysis Protocols (PRISMA-P) guidelines [19] (Additional file 1) [20]. Should there be a requirement to amend this protocol, the date and detail of each amendment will be described. The systematic review will be undertaken in accordance with the PRISMA-P statement [21] (Additional file 2).

A comprehensive systematic search for epidemiological surveys undertaken from 2000 and published up to 31 October 2023 will be undertaken in five biomedical databases: PubMed, Scopus, ProQuest, Embase, and Web of Science. The search will include grey literature and regional databases, and the reference lists from relevant studies will be hand-searched. Forward and backward citation searching will be used to identify related articles using Google Scholar. The WHO regional classification system will be used to define the countries within the WPR [22]. For each of the 37 countries within the WPR [22], the following search terms will be applied: “soil transmitted helminth*” OR STH OR *Ascaris* OR *Trichuris* OR *Nectator* OR *Ancylostoma* OR “*Strongyloides stercoralis*” OR “*Strongyloides fuelleborni”* OR hookworm* OR roundworm* OR whipworm* OR threadworm*.

### Study selection

Studies identified from the systematic search will be uploaded into Endnote X9 (Clarivate Analytics) and duplicates removed. The title and the abstracts will be independently reviewed by two authors (BG and TT) on Rayyan QCRI [23], and short-listed full text articles then evaluated against the eligibility criteria. Any disagreements in the short-listing process will be resolved through discussion, and in the event that consensus cannot be reached, dialogue will be undertaken with a third author (EG).

Studies are required to meet the following inclusion and exclusion criteria:

Inclusion criteria are as follows:

- Studies that relate to human infection and include the following STH species: *A. lumbricoides* (roundworms), *T. trichiura* (whipworms), *N. americanus*, *A. duodenale, A. ceylanicum*, *A. caninum*, and *A. braziliense* (hookworms) and *S. stercoralis* and *S. fuelleborni* (threadworms).
- Surveys with random sampling techniques that report sufficient data to facilitate the calculation of STH prevalence.
- Studies conducted within the WPR as defined by the WHO regional classification system [22].
- Where studies undertake surveys pre and post intervention regimes, only pre-intervention baseline data will be recorded. Subsequent baseline studies will identify the effectiveness of previous interventions.

Exclusion criteria are as follows:

- Case studies.
- Case series with < 10 people.
- Conference abstracts, posters, and scientific correspondence.
- Literature or systematic reviews.
- The geographic location of the survey is not provided at a higher resolution than regional level (i.e., country level reports will be excluded).
- Surveys that do not represent the general population or PSAC/SAC.
- Transient populations that do not represent the geography in which they are surveyed, e.g., recent refugee arrivals.
- Due to resource constraints, articles not published in English.

### Data extraction

Two authors (BG and TT) will independently extract data from the included studies into a Microsoft Excel (version 2016) spreadsheet. The data extraction spreadsheet will be piloted on five papers and refined if required. The proposed data extraction tool is provided in Additional file 3.

Where available, the following data will be extracted for each eligible study: first author, year of publication, year of study, study location including the name of the administrative region and longitude and latitude co-ordinates in decimal degrees format (with conversion done where required), study site (e.g., school, community), number of people screened for STH, number of people diagnosed with STH infection, species of STH, infection intensity (eggs/gram or WHO classification), diagnostic method, sample type, number of samples taken and analyzed per participant, demographic factors (age, sex), prevalence of co-infection, and name of co-infectious agent. The authors of the relevant papers will be contacted should there be a need for additional information. In the event that there are duplicate surveys for a given location, the study with the most recent and greatest amount of data will be included within the analysis.

### Methodological quality and publication bias assessment

A modified version of the Newcastle-Ottawa Quality Assessment Scale [24], Additional file 4, will be used by two authors (BG and TT) to evaluate the methodological quality of the included studies. To ensure agreement between the two researchers, the quality assessment tool will be piloted on 10 randomly selected studies, and any differences in opinion will be resolved through discussion with a third author (EG). The quality assessment (QA) scores range from 0 to 9; scores between 1 and 4 will be defined as low quality, scores between 5 and 7 will be defined as medium quality, and scores between 8 and 9 will be defined as high quality. A sensitivity analysis will be employed to evaluate the impact of methodological quality upon results of the review.

Potential publication bias and small study effects will be detected with funnel plots. Egger’s method will be utilized to evaluate asymmetry, and publication bias will be considered significant when *p* ≥ 0.05 [25].

### Covariate data sources

Covariate data for multivariable analysis will be obtained from publicly accessible records. Population data will be obtained from World Pop [26], and information on health care accessibility will be obtained from the Malaria Atlas Project (MAP) [27]. Data on climatic variables such as mean temperature, precipitation, and solar radiation will be obtained from the Global Climate Database [28]. Data on altitude will be obtained from the Shuttle Radar Topography Mission (SRTM) [29], and polygon shapefiles for the administrative boundaries of each country will be obtained from the Data-Interpolating Variational Analysis (DIVA)- Geographic Information System (GIS) [30].

### Geocoding

Extracted STH survey data will be geolocated to a specific coordinate of latitude and longitude (in decimal degrees format) where possible or the smallest polygon available otherwise (village or district). When the STH prevalence survey data are reported at a district level, coordinates of the district centroid will be used for georeferencing. Village locations will be identified using Google Maps. In instances when the STH prevalence survey has been reported at a district level (i.e., a polygon), a centroid that is spatially weighted according to population density will be used. The survey locations for each study will be stored in a geographical information system, ArcGIS (ESRI, Redlands, CA, USA). Data on STH prevalence and covariates will be linked according to a location using ArcGIS, to produce a spatially referenced dataset for analysis.

### Geospatial analysis

Bayesian model-based geostatistics (MBG) will be used to generate spatially continuous estimates of the national prevalence of each STH mapped at a resolution of 1 km^2^. Within the MBG framework, a logistic regression model will be fitted to the prevalence data using both fixed covariate effects and random spatial effects. Covariates for the spatial model will be selected using a fixed-effects logistic regression model (with an exclusion criterion of Wald *p* > 0.2). Covariates included in the model will be selected based on evidence of association with STH infection from previous studies and based on the availability of region-wide representative data. Before fitting the model, all covariates will be checked for multi-collinearity using variance inflation factors (VIF). Those variables with a VIF greater than 6 will be excluded from the final model.

Different geospatial models will be constructed independently for each species of STH. Here, we present how the model for the prevalence of a single species of STH will be constructed, but the approach will be identical for the other STH species. A Bayesian geospatial model will be fitted for the prevalence survey data that includes covariates (fixed effects) and spatial effects [31]. The proportion of cases at each surveyed location *j* will be the response variable and will be assumed to follow a binomial distribution: *Y*_*j*_~*Binomial* (*n*_*j*_, *p*_*j*_), where *Y*_*j*_ is the observed prevalence of infection, *n*_*j*_ is the number of individuals testing for infection, and *p*_*j*_ is the predicted prevalence at location *j*, with *j* = (1, …, *n*). The predicted prevalence will be associated via a logit link function to a linear predictor defined as follows:

$$\textrm{logit}\left({p}_j\right)=\log \left(\frac{p_j}{1-{p}_j}\right)=\alpha +{\sum}_{z=1}^Z{\beta}_z{\boldsymbol{X}}_{z,j}+{\zeta}_{j\kern0.5em },$$

where *α* is the intercept, *β* is a matrix of covariate coefficients, ***X*** is a matrix of *Z* covariates, and *ζ*_*j* _ is a spatial random field modelled using a Gaussian process with mean 0 and a Matérn covariance function. The covariance function will be defined by two parameters: the range *ρ*, which represents the distance beyond which correlation becomes negligible, and *σ* will be the marginal standard deviation [32, 33]. Due to the Bayesian characteristics of the geospatial model, priors need to be defined for all parameters (and hyperparameters) in the model. Non-informative priors will be used for *α* (uniform prior with bounds –∞ and ∞), and we will set normal priors with mean = 0 and precision (the inverse of the variance) = 1 × 10^−4^ for each *β*_*z*_. We will use default priors for the parameters of the spatial random field [34]. Parameter estimation will be done using the Integrated Nested Laplace Approximation (INLA) approach in R (R-INLA) [32, 33]. A relatively large number of samples (15,000 samples) will be computed to ensure that a satisfactory characterization of the posterior distribution of all parameters can be obtained.

### Prediction maps

Predictions of the prevalence of each infection at unsampled locations will be made at 1 km^2^ resolution by interpolating the spatial random effects and adding them to the sum of the products of the coefficients for the spatially variant fixed effects at each prediction location. The intercept will be added, and the overall sum will be back-transformed from the logit scale to the prevalence scale, providing prediction surfaces that show the estimated prevalence of disease for all prediction locations.

### Co-distribution

To obtain a co-endemicity map, the spatial predicted prevalence surface for each STH species will be overlaid in the GIS software. This process allows for the identification of overlapping areas where the prevalence of two, three, or four species is above a selected threshold.

## Discussion

NTDs exacerbate the disadvantage of those most commonly affected by fueling the poverty cycle [35]. Efficacious use of scare resources is pertinent for populations impacted by infection. Delivering the ambitious WHO STH 2030 targets [18] will require detailed epidemiological data to inform resource allocation and prioritization.

Geospatial meta-analysis has been used to evaluate STH prevalence in other regions [14–17] and to evaluate the distribution of other infections such as HIV [36], tuberculosis (TB) [37], malaria [38–40], cholera [41], and dengue [42]. This analysis approach enables the data from multiple surveys and their associated spatial components to be assimilated in one study [37]. Although geospatial meta-analysis provides advantages over separate analyses and increases the probability of making accurate estimates over geographical areas [37], it is acknowledged that combining discrete data sets can introduce spatial biases.

The methodology proposed for this study provides an opportunity to maximize the impact of available data whilst also highlighting data gaps. It is hoped that the results will inform regional NTD policy and help STH control programs prioritize resource allocations within the region.

### Supplementary Information

**Additional file 1.** PRISMA-P (Preferred Reporting Items for Systematic review and Meta-Analysis Protocols) 2015 checklist: recommended items to address in a systematic review protocol [20].

**Additional file 2.** PRISMA checklist of items to include when reporting a systematic review or meta-analysis [21] adapted by BioMed Central journals [43].

**Additional file 3.** Data extraction tool.

**Additional file 4.** Newcastle-Ottawa Scale adapted for cross-sectional studies (maximum total = 9 points.

## Data Availability

Not applicable.

## Abbreviations

ACE-NTD: Australian Centre for Control and Elimination of Neglected Tropical Diseases
DIVA-GIS: Data interpolating variational analysis
GIS: Geographic information system
GMRF: Gaussian Markov random field
INLA: Integrated nested Laplace approximation
MAP: Malaria atlas project
MBG: Bayesian model-based geostatistics
MDA: Mass drug administration
NHMRC: National Health and Medical Research Council
NTD: Neglected tropical disease
PCR: Polymerase chain reaction
PRISMA: Preferred reporting items for systematic review and meta-analysis
PRISMA-P: Preferred reporting items for systematic review and meta-analysis protocol
PSAC: Pre-school age children
QA: Quality assessment
SAC: School age children
SRTM: Shuttle radar topography mission
STH: Soil transmitted helminth
TB: Tuberculosis
WHO: World Health Organization
WPR: Western Pacific region
