# Correction: Protocol for spatial prediction of soil transmitted helminth prevalence in the Western Pacific region using a meta‑analytical approach

**DOI:** 10.1186/s13643-024-02495-3

**Published:** 2024-02-22

**Authors:** Beth Gilmour, Kingley Wangdi, Angela Cadavid Restrepo, Tsheten Tsheten, Matthew Kelly, Archie Clements, Darren Gray, Colleen Lau, Fe Esperanza Espino, Chona Daga, Vanessa Mapalo, Susana Vaz Nery, Adam Bartlett, Eyob Alemayehu Gebreyohannes, Kefyalew Addis Alene

**Affiliations:** 1 School of Population Health, Faculty of Health Sciences, Curtin University, Kent St, Bentley, WA, Western Australia 6102 Australia; 2 Geospatial and Tuberculosis Research Team, Telethon Kids Institute, West Perth, Western Australia Australia; 3 Australia National University, Canberra, Australia; 4 The University of Queensland, Brisbane, Australia; 5 Queen’s University, Belfast, Northern Ireland; 6 Research Institute for Tropical Medicine, Muntinlupa, Philippines; 7 The Kirby Institute, University of New South Wales, Kensington, Australia; 8 University of South Australia, Adelaide, Australia

**Correction: Syst Rev 13, 55 (2024)**

10.1186/s13643-024-02469-5

Following publication of the original article [1], the author name Susana Vaz Nery was incorrectly captured as Suzana Nashkova. The original article has been corrected.
