# Supplementary material for: Protocol for spatial prediction of soil transmitted helminth prevalence in the Western Pacific region using a meta-analytical approach
Source: Syst Rev. 2024 Feb 6;13:55. doi: 10.1186/s13643-024-02469-5 (PMC10845450; doi:10.1186/s13643-024-02469-5)
Supplement: Supplementary file 3 — Additional file 3. Data extraction tool. [file 13643_2024_2469_MOESM3_ESM.docx]

**Additional File 3:** Data extraction tool

The following headings will be used to extract data into Excel (version 2016):

- First author
- Year of publication
- Year of study/data collection
- Country
- Study location e.g., district, sub-district, village, longitude, and latitude
- Study site (e.g., school, community etc)
- Sample type(s) (e.g., blood, fecal)
- Number of samples taken and analyzed per participant
- Infectious agent(s) (e.g., *A.lumbricoides*, *T.trichuris*)
- Diagnostic method(s) (e.g., microscopy, polymerase chain reaction (PCR), serology)
- Study population (children, adult, both)
- Study population age group (<15 years; ≥15 years)
- Study population median age
- Study population size (n)
- Male (# male within the study population)
- Female (# female within the study population)
- Number of people infected
- Co-infection (name of infectious agent)
- Prevalence of co-infection (# co-infected)
- Comments/notes
