# Supplementary material for: Protocol for spatial prediction of soil transmitted helminth prevalence in the Western Pacific region using a meta-analytical approach
Source: Syst Rev. 2024 Feb 6;13:55. doi: 10.1186/s13643-024-02469-5 (PMC10845450; doi:10.1186/s13643-024-02469-5)
Supplement: Supplementary file 4 — Additional file 4. Newcastle-Ottawa Scale adapted for cross-sectional studies (maximum total = 9 points. [file 13643_2024_2469_MOESM4_ESM.docx]

**Additional File 4:** Newcastle-Ottawa Scale adapted for cross-sectional studies (maximum total = 9 points)

|  | |
| --- | --- |
| **Study Population** | |
| 1 | The study population is clearly defined |
| 0 | The study population is not clearly defined |
| **Representativeness of the sample** | |
| 2 | Study sample is representative of the study population (all subjects or random sampling) |
| 1 | Study sample comprises a select group of the study population (non-random sampling) |
| 0 | No description of the sampling strategy. |
| **Ascertainment of specimen collection methods** | |
| 1 | The study clearly defines specimen collection methodologies |
| 0 | The study does not detail specimen collection methodologies |
| **Sample size** | |
| 1 | Justified and satisfactory (sample size and power calculation included) |
| 0 | Not justified |
| **Non-respondents** | |
| 1 | Comparability between respondents and non-respondent’s characteristics are established |
| 0 | No description of the response rate or the characteristics of the responders and the non-responders. |
| **Comparability:** | |
| **Impact of Bias (selection bias, measurement bias, participant reporting, confounders)** | |
| 1 | Where relevant, the study acknowledges and mitigates for potential bias. When comparisons are made between different study populations results are adjusted for confounders |
| 0 | Where appropriate, the study does not acknowledge or mitigate for potential bias. When comparisons are made between different study populations results are not adjusted for confounders |
| **Assessment of the outcome (STH infection)** | |
| 1 | Objective diagnostic methodology with units of measurement and /or definitions |
| 0 | No definitive diagnosis or self-report |
| **Statistical analysis** | |
| 1 | The statistical method used is clearly described and appropriate for the analysis undertaken. Where comparisons are made between population groups, the measurement of the association is presented, including confidence intervals and the probability level (p value) |
| 0 | The statistical method is inappropriate/not described/incomplete |
